# 1-(Acylamino)alkylphosphonic Acids—Alkaline Deacylation

**DOI:** 10.3390/molecules23040859

**Published:** 2018-04-09

**Authors:** Marek Cypryk, Jozef Drabowicz, Bartlomiej Gostynski, Marcin H. Kudzin, Zbigniew H. Kudzin, Pawel Urbaniak

**Affiliations:** 1 Centre of Molecular and Macromolecular Studies, Polish Academy of Sciences, Sienkiewicza 120a, Lodz 90-363, Poland; mcypryk@cbmm.lodz.pl (M.C.); bgostyns@cbmm.lodz.pl (B.G.); 2 Department of Chemistry and Environment Protection, Jan Dlugosz University, Czestochowa 42-200, Poland; 3 Textile Research Institute, Brzezinska 5/15, Lodz 92-103, Poland; kudzin@iw.lodz.pl; 4 Faculty of Chemistry, University of Lodz, Tamka 12, Lodz 91-403, Poland; pawelurb@chemia.uni.lodz.pl

**Keywords:** amino acids, aminophosphonic acids, 1-aminoalkylphosphonic acids, 1-(acylamino)alkylphosphonic acids, ^31^P NMR spectra of intermediates, hydrolytic deacylation

## Abstract

The alkaline deacylation of a representative series of 1-(acylamino)alkylphosphonic acids [(AC)-AA^P^: (AC) = Ac, TFA, Bz; AA^P^ = Gly^P^, Ala^P^, Val^P^, Pgl^P^ and Phe^P^] in an aqueous solution of KOH (2M) was investigated. The results suggested a two-stage reaction mechanism with a quick interaction of the hydroxyl ion on the carbonyl function of the amide R-C(O)-N(H)- group in the first stage, which leads to instant formation of the intermediary acyl-hydroxyl adducts of R-C(O^−^)_2_-N(H)-, visible in the ^31^P NMR spectra. In the second stage, these intermediates decompose slowly by splitting of the RC(O^−^)_2_-N(H)- function with the subsequent formation of 1-aminoalkylphosphonate and carboxylate ions.

## 1. Introduction

Aminoalkylphosphonic acids (AA^P^) are structural analogues of amino acids (AA^C^) [1], some of which are of natural origin [2]. Due to the structural analogy, they present similar biological properties to the class and are important inhibitors of enzymes of the amino acids metabolism [1,3] (Table 1).

Several papers reflected the complexing abilities of this class of compounds [16,17,18] and their pharmacological [19,20,21,22], agro-chemical [23] and industrial [11,24,25] applications. Therefore, the studies on the synthesis [26,27,28,29,30] and physico-chemical properties [31,32,33,34,35,36,37,38,39,40] of AA^P^ and their derivatives constitute an important topic in chemistry and biochemistry also in addition to material science (e.g., Self-assembled monolayers agents, SAMs [41]). 

The 1-(acylamino)alkylphosphonic acids (AC)-AA^P^ belong to the interesting group of compounds that is of potential pharmacological importance since the corresponding 1-*N*-aminoacylamino derivatives of AA^C^-AA^P^ (mixed *P*-terminal phosphono-dipeptides, e.g., Alaphosphaline) exhibit antibacterial activity [1,23] (Table 1). 

**Figure 1:**
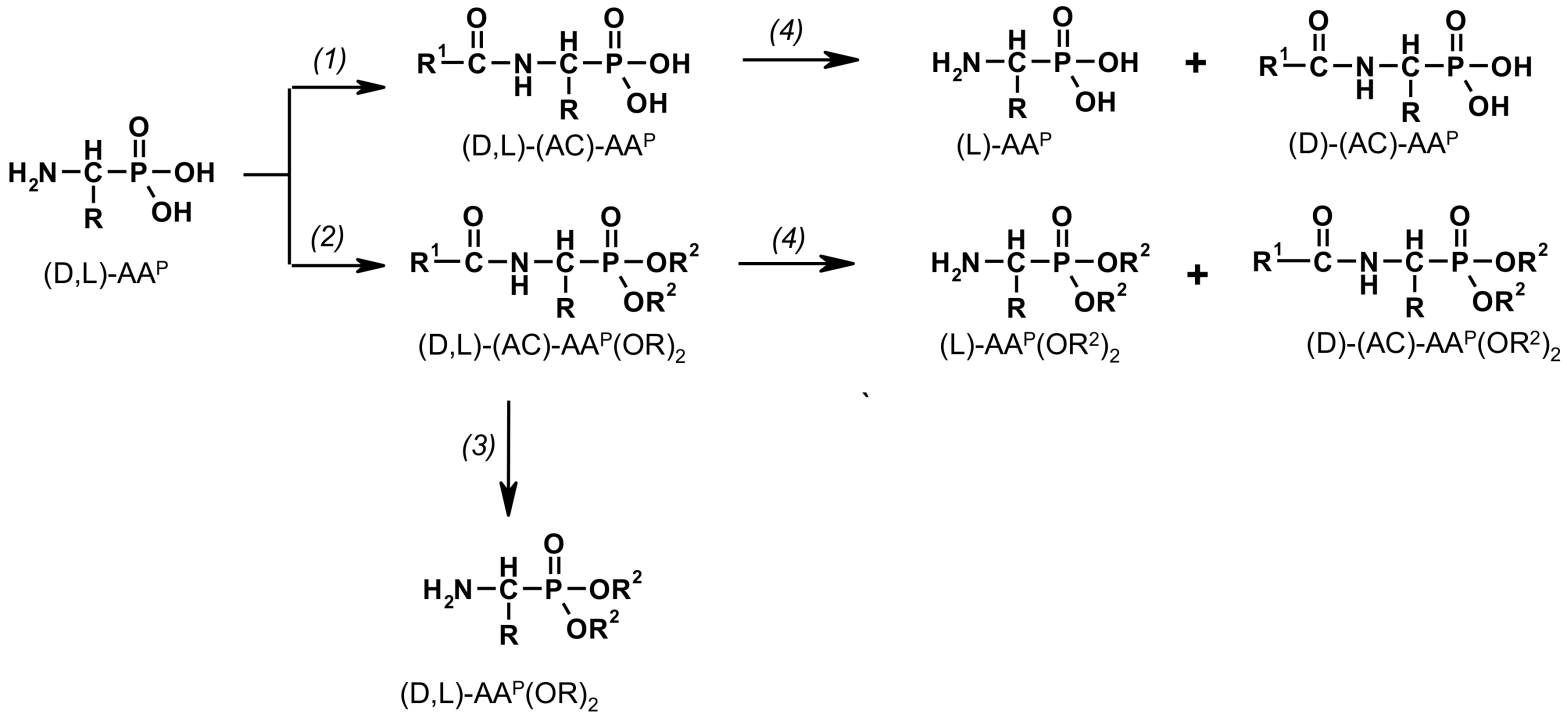
Transformation of 1-aminoalkylphosphonic acids via *N*-acyl-derivatives [(*1*) R^1^-C(O)OH/[R^1^-C(O)]_2_O [36,37,38]; (*2*) R^1^-C(O)OH/[R^1^-C(O)]_2_O/HC(OR^2^)_3_ [42]; (*3*) NaBH_4_/MeOH [43]; (*4*) enzymatic hydrolysis of racemic (D,L)-(AC)-AA^P^ and (D,L)-(AC)-AA^P^(OR)_2_ [44,45,46,47,48]].

**Figure 2:**
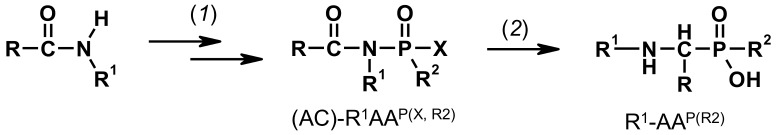
Scheme of Engelmann-Pikl-Oleksyszyn methods for synthesis of secondary 1-aminoalkylphosphonic (R^2^=OH) and 1-aminoalkylphosphinic (R^2^=H, alkyl) acids (*1*) aldehyde, R-PCl_2_/AcOH; and (*2*) 5M HCl-H_2_O, Δ8–10 h.

1-(Acylamino)alkylphosphonic acids [36,37] and esters [42] are easily formed from starting aminoalkylphosphonic acids, the latter being substrates for selective deacylation to *O*,*O*-dialkyl aminoalkylphosphonates [43]. Both of these groups are substrates for stereoselective enzymatic hydrolysis [44,45,46,47,48] (Figure 1). 1-(Acylamino)alkylphosphonic acids are also formed in so-called Engelman-Pikl-Oleksyszyn method synthesis of 1-aminoalkylphosphonic and 1-aminoalkylphosphonic acids, which starts from appropriate amides and phosphorus chlorides [10,27,49,50] ( Figure 2). As some secondary 1-aminoalkanephosphonates are unstable in the process of acidic degradation [40], alkaline hydrolysis can present an alternative method in such procedures.

The susceptibility of (AC)-AA^P^ to hydrolysis/solvolysis constitutes an important factor influencing their biological activity, especially during penetration through cell membranes. Therefore, the detailed studies on deacylation of these compounds should be helpful in obtaining a deeper understanding of this phenomenon. To tackle this problem, we have decided to carry out studies devoted to deacylation of (AC)-AA^P^ under different reaction conditions. Our preliminary results of experiments were carried out in aqueous media in the pH range of 0–6.5, which occurs namely in an aqueous 2M HCl and buffer solutions. These are briefly described in a recently published article [51]. As an obvious and necessary continuation of this topic, this paper presents our results on the deacylation of representative types of 1-(acylamino)alkylphosphonic acids (AC)-AA^P^ in aqueous 2M KOH solution. These include 1-(acetylamino)alkylphosphonic Ac-AA^P^, 1-(trifluoroacetylamino)alkylphosphonic acids TFA-AA^P^ and 1-(benzoylamino)alkylphosphonic Bz-AA^P^, derived from representative 1-aminoalkylphosphonic acids AA^P^ (Gly^P^, Ala^P^, Val^P^, Pgl^P^ and Phe^P^). 

## 2. Results and Discussion

It is generally known that amides can be hydrolyzed with either acidic or basic catalysis, with products being the free acid and the ammonium/substituted ammonium ions or the salts of the acid and ammonia/amine, respectively. Both the acid- and base-catalyzed hydrolyses are essentially irreversible, since salts are formed in both cases [52,53,54,55]. Water alone is not sufficient to hydrolyze most of amides [56]. The very low rate of amide hydrolysis by water has been measured by Kahne & Still [57]. 

A kinetic study has been conducted on the alkaline hydrolyses of several types of amides, including formanilides [58,59], *N*-methylformanilides and *N*-acetanilides [60], 1,8-bis(trifluoroacetylamino)-naphthalene [61] also in addition to *N*-methylacetamide, *N*-methylbenzamide and acetanilide [62]. A mild protocol for the alkaline hydrolysis of secondary and tertiary amides in non-aqueous conditions at room temperature or under reflux has been recently described [63].

The structural analogy of 1-(acylamino)alkylphosphonic acids (AC)-AA^P^ and 1-(acylamino)-alkanoic acids (AC)-AA^C^ or amides causes the mechanism of hydrolytic scission of the amide linkage R-C(O)-N in common amides and in 1-(acylamino)alkylphosphonic acids. These should be ruled by similar if not the same mechanisms.

### 2.1. Investigations on the Deacylation Course of 1-(acylamino)alkylphosphonic Acids

The (AC)-AA^P^ contain amidic functions, which has a hydrolytic sensitivity that should be dependent on the structure of their acyl moieties and on the type of applied hydrolytic medium [49]. For conducting an inquiry into the hydrolytic stability of (AC)-AA^P^, we undertook deacylation investigations of these compounds in aqueous 2M KOH solutions.

**Figure 3:**
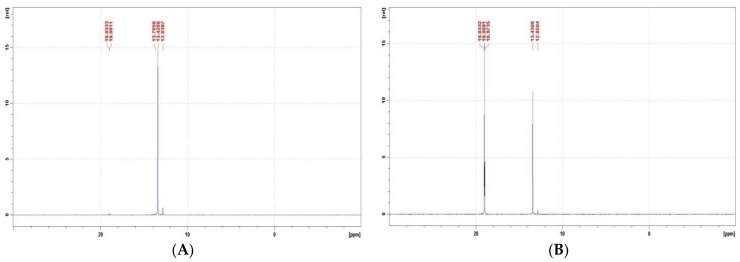
^31^P NMR spectra of Ac-Gly^P^ recorded in 2 M KOH. (**A**) Immediately after mixing the reaction mixture, and (**B**) After 192 h of exposition of the reaction mixture. [Ac-Gly^P^: 13.4 ppm; Gly^P^: 19.0 ppm].

**Figure 4:**
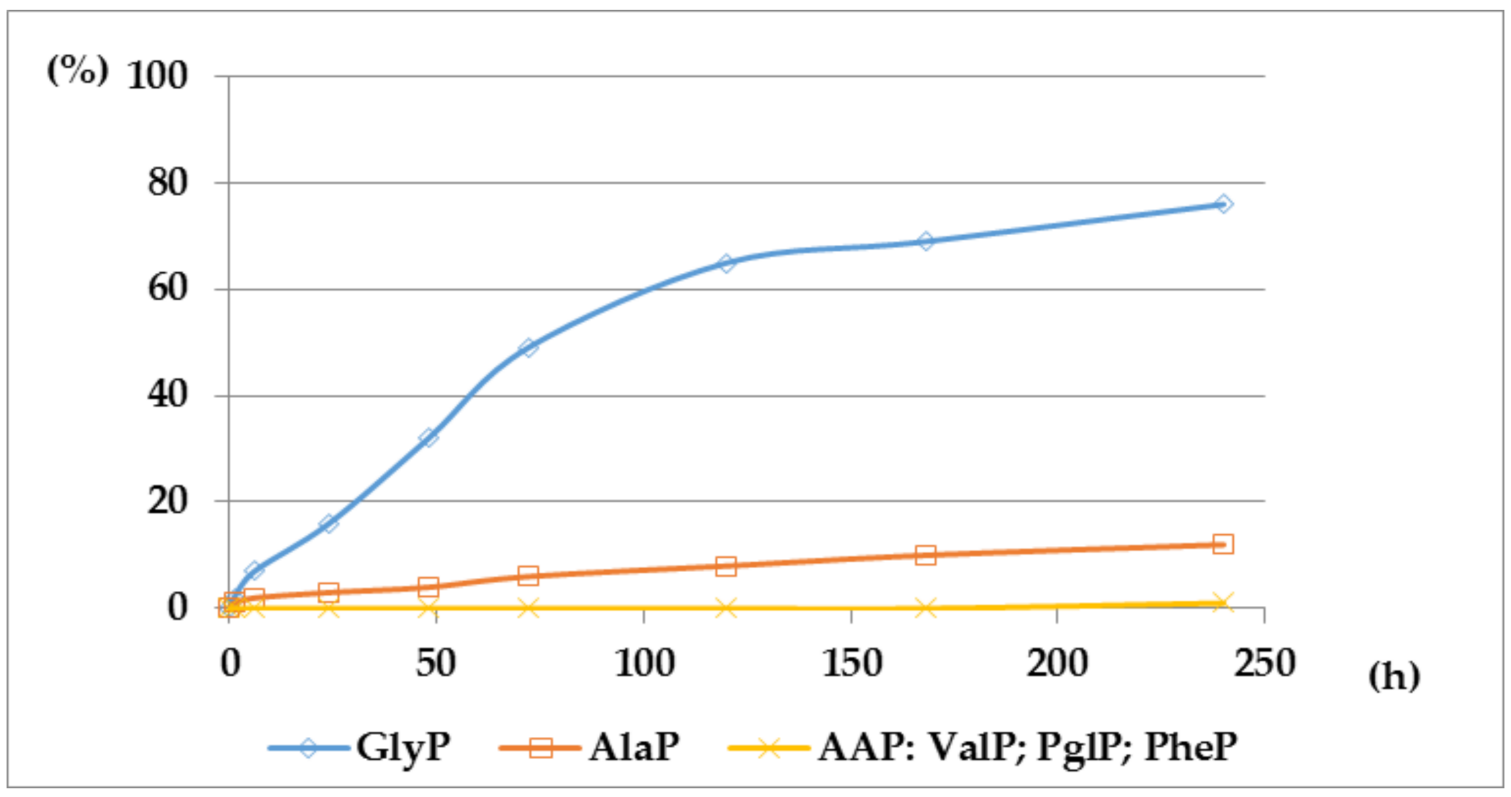
A solution of 0.1 M 1-(acetylamino)alkylphosphonic acids Ac-AA^P^ in 2 M KOH reacted at 25 °C, which was monitored by ^31^P NMR [DD (%) vs. time (h)].

**Figure 5:**
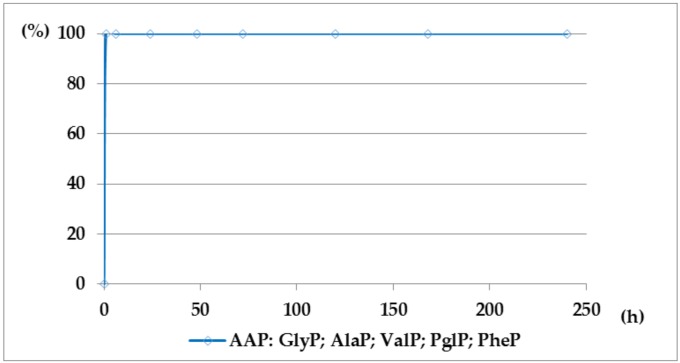
A solution of 0.1 M 1-(trifluoroacetylamino)alkylphosphonic acids TFA-AA^P^ in 2 M KOH reacted at 25 °C, which was monitored by ^31^P NMR [DD (%) vs. time (h)].

**Figure 6:**
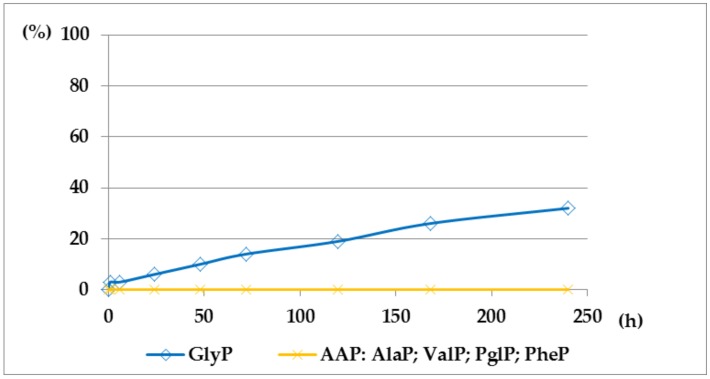
A solution of 0.1 M 1-(benzoylamino)alkylphosphonic acids Bz-AA^P^ in 2 M KOH reacted at 25 °C, which was monitored by ^31^P NMR [DD (%) vs. time (h)]. Thus, the presented graphs illustrate much higher stability of benzoyl-derivatives Bz-AA^P^ (Bz-Gly^P^: DD_200h_ ~30%; for Bz-Ala^P^ and Bz-Pgl^P^: DD_200h_ < 3%) in comparison with acetyl-derivatives Ac-AA^P^ (Ac-Gly^P^: DD_200h_ ~70%; for Ac-Ala^P^: DD_200h_ ~20%; and Ac-Pgl^P^: DD_200h_ < 7%).

The application of ^31^P NMR monitoring of the reaction course enabled identification and quantification of phosphoric components of the reaction mixtures formed. Essentially, monitoring of the deacylation course of Ac-Gly^P^ in 2M KOH solution reveals the formation of Gly^P^ (Figure 3). The results of ^31^P NMR monitoring of the deacylation of the various types of (AC)-AA^P^ (Ac-AA^P^, Bz-AA^P^ and TFA-AA^P^) at a temperature of 25 °C and exposition period up to 240 h are presented in Figure 4 (Ac-AA^P^), Figure 5 (TFA-AA^P^) and Figure 6 (TFA-AA^P^), respectively. 

**Figure 7:**
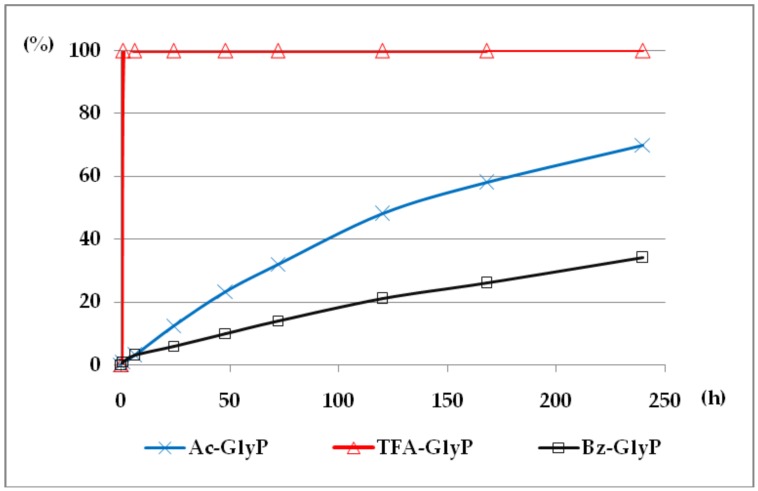
A solution of 0.1 M 1-(acylamino)methylphosphonic acids (AC)-Gly^P^ (AC: Ac, TFA, Bz) in 2M KOH reacted at 25 °C, which was monitored by ^31^P NMR [DD (%) vs. time (h)]. Points on the plots corresponds to Gly^P^ released from the parent (AC)-Gly^P^, which means Gly^P^ from Ac-Gly^P^, Gly^P^ from Bz-Gly^P^ and Gly^P^ from TFA-Gly^P^.

The comparison of hydrolytic stability of TFA-Gly^P^, Ac-Gly^P^ and Bz-Gly^P^ in 2M KOH is presented in Figure 7.

**Scheme 1:**
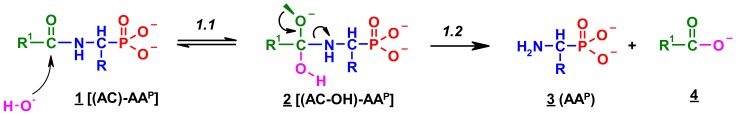
Hypothetical mechanism of deacylation of (AC)-AA^P^ in 2 M KOH solution.

Since no products of the scission of the C-P bond in all investigated cases were observed, the deacylation mechanism of (AC)-AA^P^ in alkaline solutions can be illustrated by an analogy to the corresponding basic deacylation of 1-(acylamino)alkanoic acids (Scheme 1) [54,62,63]. 

This includes the prior interaction of hydroxyl ion on the carbonyl carbon of **1**, which occurs with the formation of the tetrahedral intermediate state **2** (Scheme 1). These intermediates were observed on ^31^P NMR spectra as signals down-shifted in comparison with the parent signals of AC-AA^P^ (e.g., Figure 2). The ^31^P NMR chemical shifts of (AC)-AA^P^ (**1**) and the corresponding adduct compounds AC(OH)-AA^P^ (**2**) formed are listed in Table 2.

**Figure 8:**
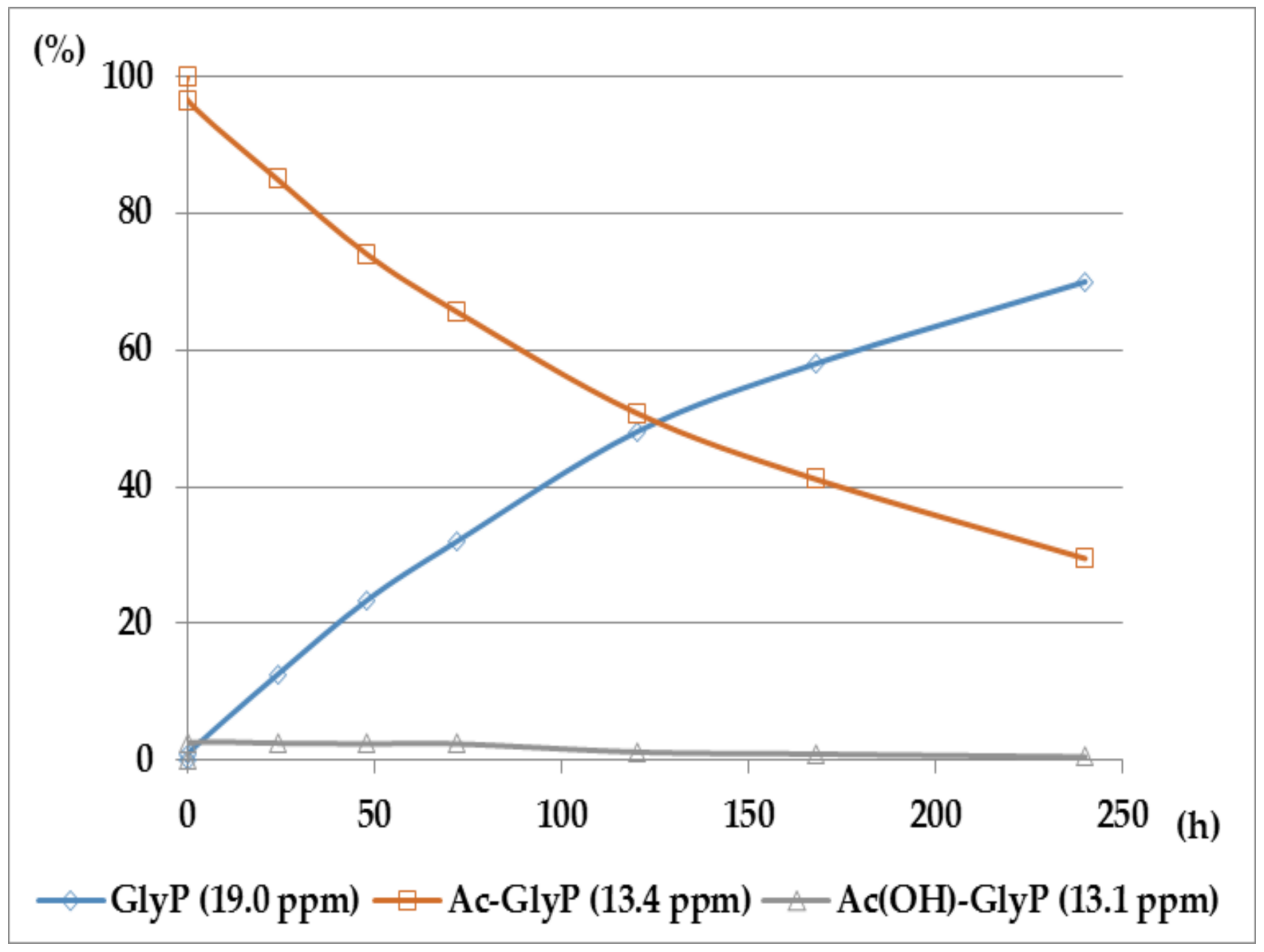
Ac-Gly^P^ hydrolysis in 2M KOH [DD (%) vs. time (h)].

These compounds [AC(OH)]-AA^P^] slowly disappeared during the deacylation progress (Figure 8).

The rehybridization (sp^3^→sp^2^) of the carbonyl carbon in **2** enforces the splitting of the amide bond R-C(O)-N and the subsequent formation of the aminophosphonate **3**. 

**Figure 9:**
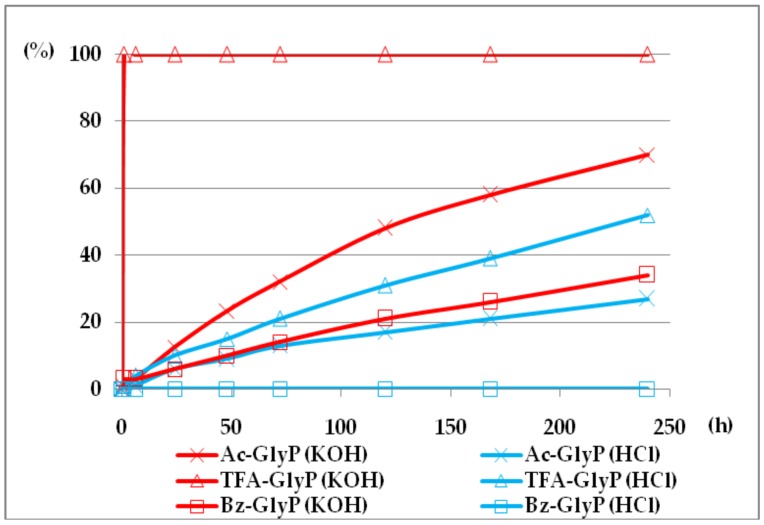
(AC)-Gly^P^(Ac-Gly^P^, TFA-Gly^P^, TFA-Gly^P^) hydrolysis in aqueous solutions of 2M KOH and 2M HCl [DD (%) vs. time (h)].

The comparison of (AC)-AA^P^ stability in aqueous 2M KOH and 2M HCl solutions is given in Figure 9.

### 2.2. Quantum Chemical Calculations for Deacylation of (AC)-AA^P^ in Basic Conditions

**Figure 10:**
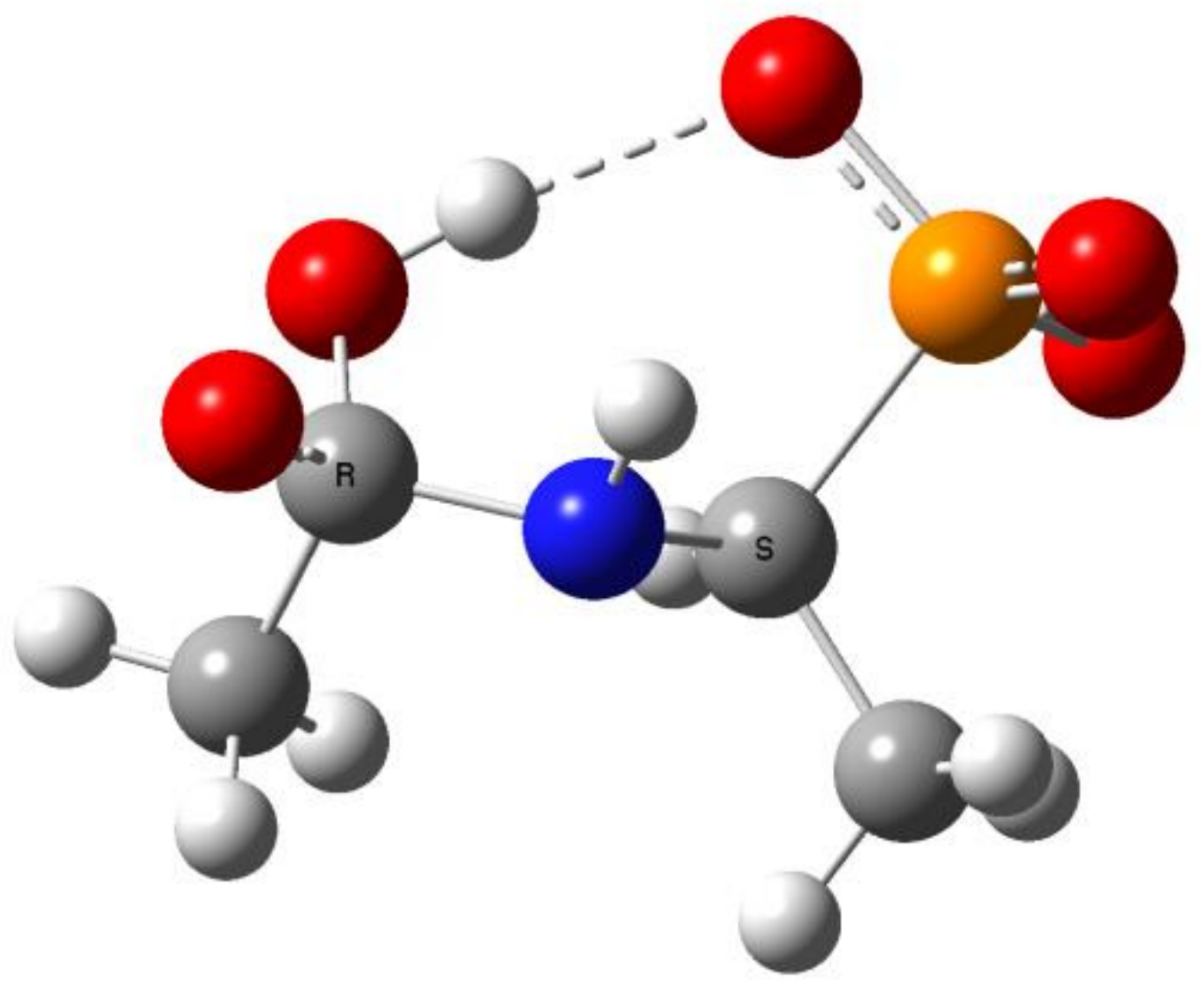
The structure of the addition product **2** (R=R^1^=Me, Ac(OH)-Ala^P^) with intramolecular hydrogen bond between OH and phosphoryl group (orange—phosphorus, blue—nitrogen, red—oxygen).

The reaction illustrated in Scheme 1 was divided into two stages (reactions *1.1* and *1.2*, respectively), which were studied separately. Stage *1.1* concerns the addition of hydroxide to amidophosphonate anion **1** with the formation of trianion **2** (the example structure of the addition product **2** for Ac-Ala^P^ (R=R^1^=Me) is given in Figure 10). Stage *1.2* involves dissociation of the trianion **2** into aminophosphonate dianion **3** and carboxylate anion **4**. We assumed that the transfer of proton from OH to the amide nitrogen in **2** proceeds simultaneously with dissociation. The thermodynamics of this two-stage process was studied by density functional theory (DFT) methods in the gas phase and in aqueous solution. Gas phase calculations were performed only for comparison with the aqueous system, which was the real reaction system to illustrate the essential role of the solvation effect.

**Stage 1:** Hydroxide anion attack on the carbonyl moiety of (AC)-AA^P^ dianion **1** (Scheme 1, r. *1.1*).

This reaction stage presumably proceeds by the attack of a lone pair of negatively charged oxygen atoms on sp^2^ carbon in the carbonyl group. This process is highly unfavorable since it involves the reaction of two anions with the formation of the molecule with a total charge of −3. The energy of this reaction in the gas phase is very high, so this process seems to be thermodynamically very unlikely (Table 3). 

However, when carried out in aqueous solutions, this reaction is much less unfavorable due to the stabilization of ionic structures by the polar solvent (Table 2). Similarly, this reaction should also be facilitated by counterions, although these were omitted in order to keep the model system simple. The values of the Gibbs free energy ΔG^298^ (kcal/mol) for reaction *1.1* in gas phase and in aqueous phase are listed in Table 3 and Table 4, respectively.

The free energy barriers, ΔG^‡^ (298 K), calculated for reaction *1.1* in a water solution are shown in Table 5. The lowest energy barriers for OH^−^ addition (Scheme 1, r. *1.1*) occur when R^1^ = CF_3_, which results from stabilization of the negative charge by the electron-withdrawing CF_3_ group. 

**Stage 2:** Dissociation of trianion **2** into aminophosphonate dianion **3** and carboxylate anion **4** (Scheme 1, reaction *1.2*).

**Figure 11:**
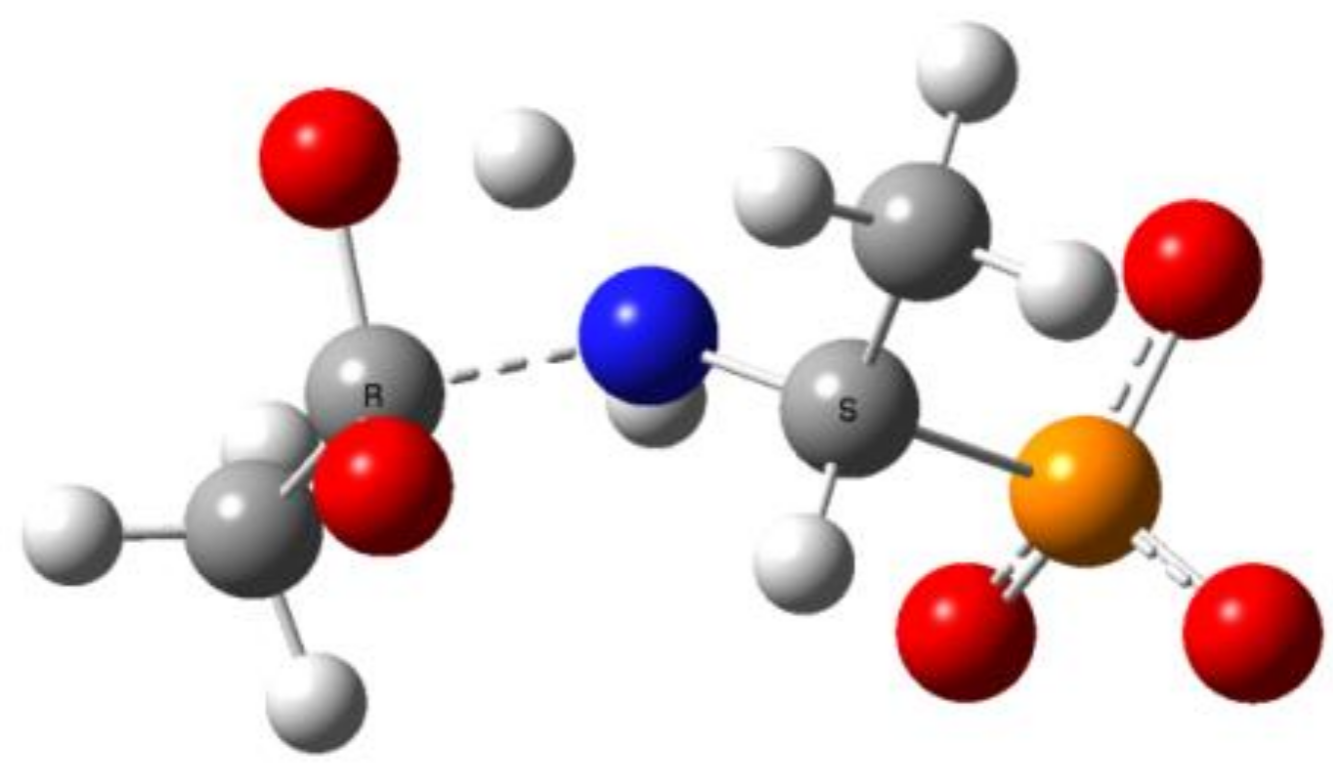
The transition state for proton transfer/dissociation of the C-N bond in Ac(OH)-Ala^P(3−)^.

This reaction stage presumably involves the proton transfer from the geminal hydroxyl group to nitrogen with dissociation of the C-N bond. This hypothesis is supported by the transition structures found for this reaction. The example of this structure for the proton transfer/dissociation of the C-N bond in Ac-Ala^P(3−)^ is shown in Figure 11. Vibrational analysis indicates that the imaginary vibration is associated with proton transfer from OH to NH. In the structure of the transition state shown in Figure 11, the geminal C-O bond distance is 1.284 Å, which corresponds well to C=O double bond. Furthermore, the C-N distance is 1.802 Å compared to 1.503 Å in the starting amide, which indicates that the bond breaking in the transition state is significantly advanced.

The Gibbs free energies for the dissociation reaction *1.2* in the gas phase and in water are presented in Table 6 and Table 7, respectively.

Comparing the thermodynamic quantities in Table 3 and Table 6, it is evident that the total free energy of the reaction *1* is negative in all cases by approximately −30 kcal/mol and thus, the entire process *1* is thermodynamically feasible. However, the very high energy barrier for the first step of reaction *1.1* (Table 3) prevents this reaction from proceeding in the gas phase.

Analogously, the reaction 1 in water is equally thermodynamically favorable (ΔG = −20 to −30 kcal/mol, Table 4 and Table 7), but the energy barrier associated with the first step *1.1* is much lower in this case (Table 4). Table 8 presents the results of free energy barrier calculations for reaction *1.2* in water.

**Figure 12:**
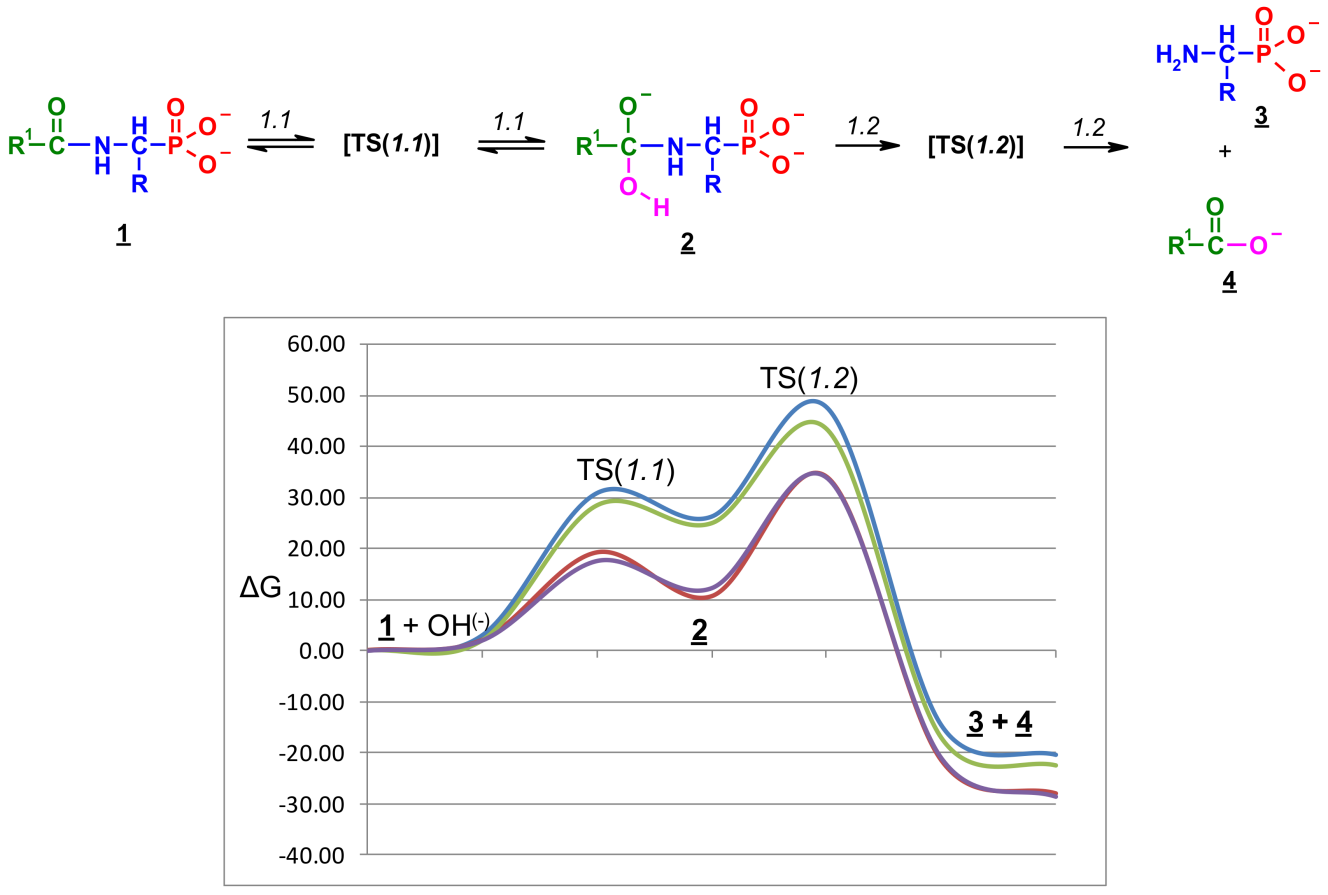
Free energy profiles ΔG^298^ (kcal/mol) for the reaction of Ac-Ala^P(2−)^ (blue), TFA-Ala^P(2−)^ (red), Bz-Pgl^P(2−)^ (green), and TFA-Pgl^P(2−)^ (purple) with hydroxyl ions in water solution (the general reaction scheme is given above).

The total Gibbs free energy profiles of entire process *1* for two selected pairs of 1-(acylamino)alkylphosphonic acids [Ac-Ala^P(2−)^, TFA-Ala^P(2−)^ , Bz-Pgl^P(2−)^ and TFA-Pgl^P(2−)^] in water solution are shown in Figure 12. 

The total Gibbs free energies and reaction barriers for the entire process *1* are shown in Table 9. Moreover, Figure 12 reveals that the energy profiles for both TFA-substituted phosphonic acids are essentially identical and all stationary points are significantly lower in energy than Ac-substituted phosphonic acids.

Therefore, the presence of a TFA group is the only factor determining the free energy barrier of the reaction. From the energy profile, it is obvious that the overall energy barrier in water ΔG^‡^ is G^298^[TS (*1.2*)] − (G^298^(**1**) + G^298^(OH^−^)), while the net Gibbs energy effect for the reaction in water solution can be calculated as ΔG_r_^298^ = G^298^(**3**) + G^298^(**4**) −[(G^298^(**1**) + G^298^(OH^−^)] = ΔG^298^(*1.1*) + ΔG^‡^(*1.2*).

The total energetic effect of the process *1* in water is negative, which means that the overall reaction is thermodynamically feasible (Table 8). The free energy of the process when R^1^=CF_3_ is also approximately 8 kcal/mol more favorable than for R^1^=Me. The comparison of values in Table 9 also reveals that the transformation *1* of substrates with R^1^=Ph are thermodynamically more favored by approximately 3 kcal/mol than R^1^=Me. Thus, the thermodynamic effect of reaction *1* depends on the R^1^ substituents in the order of: CH_3_ < Ph < CF_3_. Table 9 also leads to the conclusion that the substrates with a trifluoromethyl group in the R^1^ position systematically show free energy barriers that are approximately 10–15 kcal/mol lower than those for other substituents R^1^, due to the additional stabilization of the trianion **2** by the inductive effect of the strongly electron-withdrawing CF_3_ group. This suggests that trifluoromethyl-substituted anions should be more reactive than the other derivatives.

## 3. Materials and Methods

### 3.1. General Information

The ^31^P NMR spectra were recorded on a Bruker AV 200 spectrometer (Rheinstetten, Germany) operating at 81.01 MHz and on a Bruker Avance III 600 spectrometer (Bruker BioSpin, Rheinstetten, Germany) operating at 242.9 MHz. The1H NMR spectra were recorded on a Bruker Avance III 600 spectrometer operating at 600 MHz. The positive chemical shift values of ^31^P were reported for compounds that were absorbing at lower fields than H_3_PO_4_. The purity of the 1-aminoalkylphosphonic acids and corresponding 1-(acylamino)alkylphosphonic acids were determined by pH-metric titration using a computer aided automatic titrator connected to the EMU-meter (Wroclaw University of Science and Technology, Wrocław, Poland), which was fitted with a combined glass microelectrode Crison 5028.

### 3.2. Investigated Compounds

Phosphonoglycine (Gly^P^) was obtained according to a previous study [64]. The 1-Aminoalkylphosphonic acids of phosphonoalanine (Ala^P^); phosphonovaline (Val^P^); phosphonophenylglycine (Pgl^P^) and phosphonophenylalanine (Phe^P^) have been prepared according to a previous study [65]. 1-(Acetylamino)alkylphosphonic acids (Ac-AA^P^: Ac-Gly^P^; Ac-Ala^P^; Ac-Val^P^; Ac-Pgl^P^ and Ac-Phe^P^) and 1-(benzoylamino)alkylphosphonic acids (Bz-AA^P^: Bz-Gly^P^; Bz-Ala^P^; Bz-Val^P^; Bz-Pgl^P^ and Bz-Phe^P^) were synthesized according to a previous study [36]. 1-(Trifluoroacetylamino)alkylphosphonic acids (TFA-AA^P^: TFA-Gly^P^; TFA-Ala^P^; TFA-Val^P^; TFA-Pgl^P^ and TFA-Phe^P^) were synthesized according to a previous study [37]. (Structures, names and abbreviations of aminophosphonic acids and corresponding 1-(acylamino)phosphonic acids discussed in this work are given in Table 10). Purity of all (AC)-AA^P^ synthesized was determined by ^31^P NMR and ^1^H NMR in addition to potentiometric titration. Analytical standards (fixanals) and other reagents were purchased from Aldrich–Sigma (Poznań, Poland). 

### 3.3. Reaction of Deacylation of 1-(acylamino)alkylphosphonic Acids 

A sample of (AC)-AA^P^ (0.5 mmol) was placed into 5-mL V-vials, before being dissolved in 5 mL of appropriate solution fortified with D_2_O (2 M KOH). The vials were maintained at 25 °C ± 0.2 °C. At various time intervals, the vials were removed from the baths and the samples of 0.5 mL were taken for ^3l^P NMR analysis. The concentration of the substrate and formed products were determined from the integration of its NMR signal.

### 3.4. Computational Methods

All quantum mechanical calculations were performed using the Gaussian 09 suite of programs [66]. Geometries of model compounds in the gas phase and in water solution were optimized using the B3LYP hybrid functional containing three-parameter Becke (B3) exchange and Lee-Yang-Parr (LYP) correlation functional and the 6–31+G(d) basis set. All stationary points were identified as stable minima by frequency calculations. The vibrational analysis also provided the thermal enthalpy and entropy corrections at 298 K within the rigid rotor/harmonic oscillator/ideal gas approximation. Computed frequencies were scaled by a factor of 0.98. Transition states were verified by the intrinsic reaction path (IRC) procedure to confirm if they did link the substrates and products along the reaction path.

Free energies of the reaction in water were calculated within a continuum (implicit) solvent approximation using the Conductor-like Polarizable Continuum Model (CPCM) with UFF (Universal Force Field) cavities (SCRF = CPCM option as defined in Gaussian 09 program) [67]. This approximation assumes that molecule of the solute is placed in a cavity within the solvent, which is treated as a dielectric continuum (self-consistent reaction field). Thermodynamic functions in solution were calculated based on vibrational analysis, which was described above.

## 4. Conclusions

The analysis of ^31^P NMR results presented above leads to the following conclusions:

- The alkaline deacylation of (AC)-AA^P^ occurs through the hydroxyl adduct intermediates, which was observed on ^31^P NMR spectra of the reaction mixtures;
- The deacylation ability of investigated (AC)-AA^P^ derivatives exhibited strong dependence of electron-acceptor character of the acyl group, with CF_3_-C(O)- > CH_3_-C(O)-;
- The 1-(acylamino)alkylphosphonic acids are present in aqueous solutions with substantial stability at ambient temperatures, which decreases with temperature elevation;
- The deacylation of (AC)-AA^P^ increases substantially in basic (2 M KOH) solutions;
- The lowest deacylation ability (highest stability) was found for the 1-(benzoylamino acids Bz-AA^P^;
- For the same type of 1-(acylamino)-derivatives (AC)-AA^P^, the highest stability was found in(AC)-Val^P^ and (AC)-Phe^P^; lower stability in (AC)-Ala^P^; and the lowest in (AC)-Gly^P^ and (AC)-Pgl^P^;
- All examined ^31^P NMR spectra of deacylation mixtures did not reveal any trace of H_3_PO_3_ and/orH_3_PO_4_, which represent the products of dephosphonylation or oxidative dephosphonylation of (AC)-AA^P^.

The theoretical calculations lead to the following conclusions:

The substrates with the trifluoromethyl group in R^1^ position are predicted to be significantly more reactive than the other phosphonic amino acids studied, which is consistent with the experimental results. The intermediates and products when R^1^=CF_3_ are also more thermodynamically favored than the other derivatives. This difference in reactivity is due to additional stabilization of the trianion **2** by the inductive effect of the strongly electron-withdrawing CF_3_ group. The calculations did not provide unequivocal information about relative reactivities of other AA derivatives as the calculated reaction energy barriers are similar (within a computational error), which is shown in Table 9 and Figure 12. The calculation results are consistent with those reported for acidic deacylation of 1-(acylamino)alkylphosphonic acids [51]. As CF_3_ is strongly electron-withdrawing, this group plays an activating role in basic media, increasing the acidity of carbonyl moiety. In contrast, it has a deactivating effect in acidic media, decreasing the basicity of carbonyl oxygen.

## Figures and Tables

**Table 1:** Structures of aminophosphonic acids (AA^P^)—phosphonic analogs of amino acids (AA^C^) and 1-(acylamino)alkylphosphonic acids (AC)-AA^P^ and phosphonopeptides AA^C^-AA^P^ and AA^P^-AA^C^ [4,5,6,7,8,9,10,11,12,13,14].

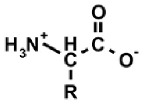	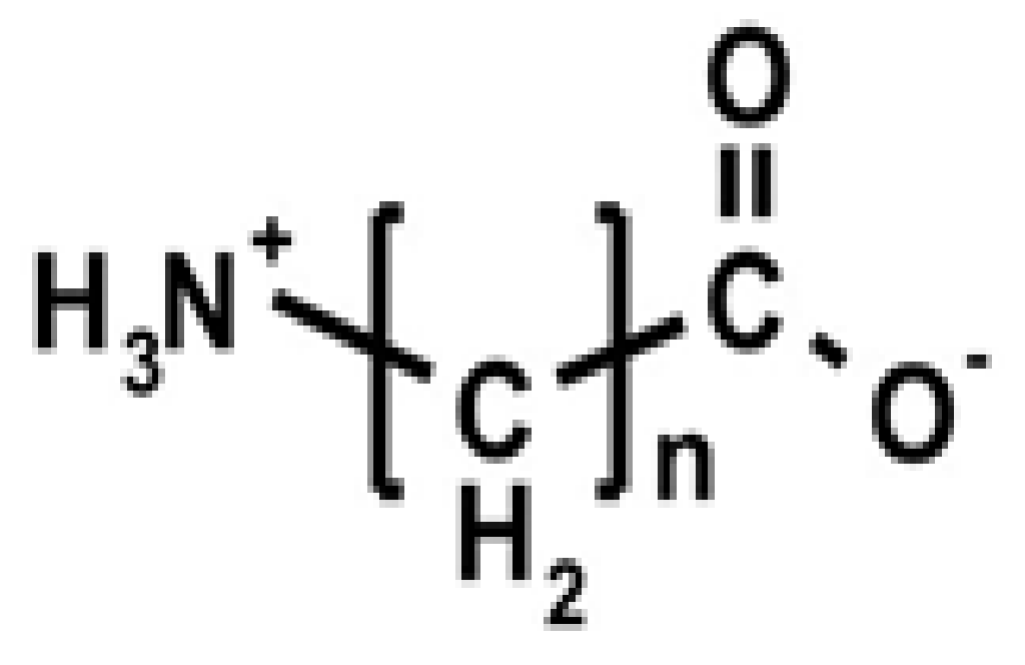	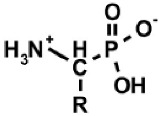	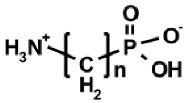	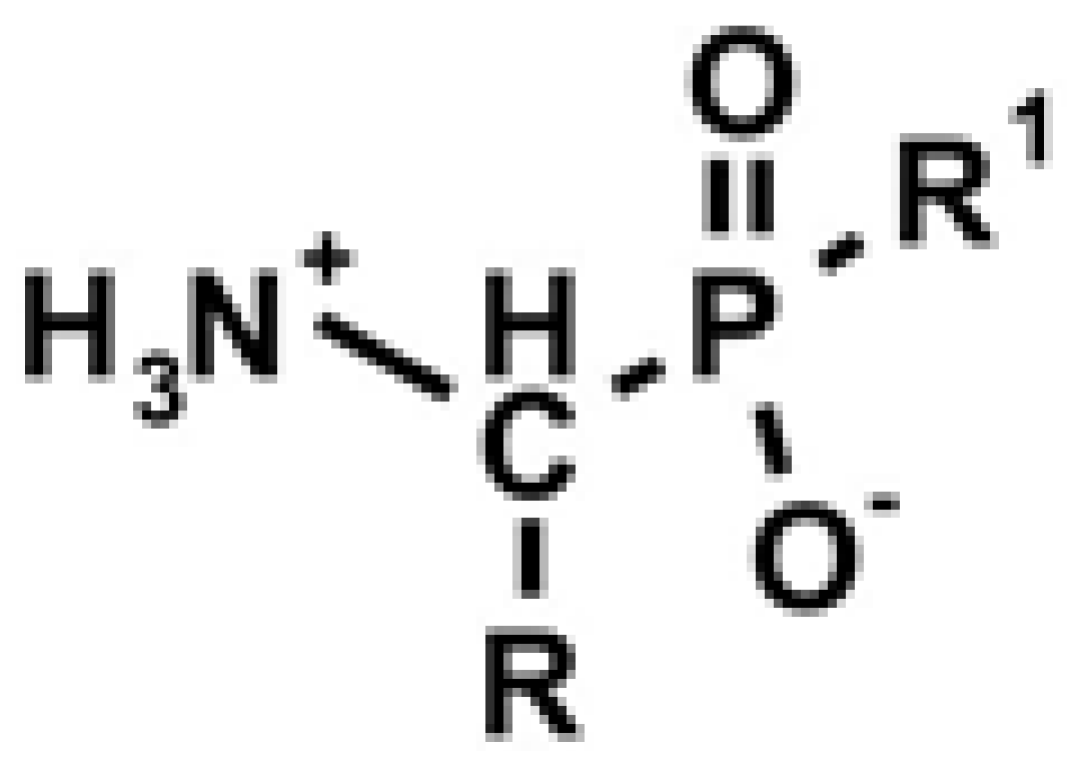
**AA^C^**	ω-AA^C^	AA^P^	ω-AA^P^	AA^P–R^
**Naturally occurring aminophosphonic acids.**				
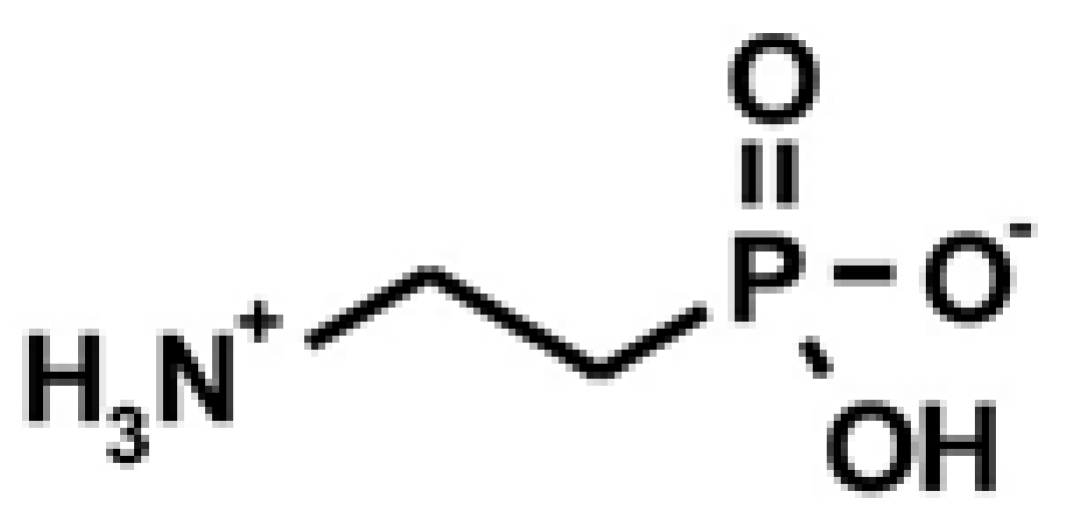	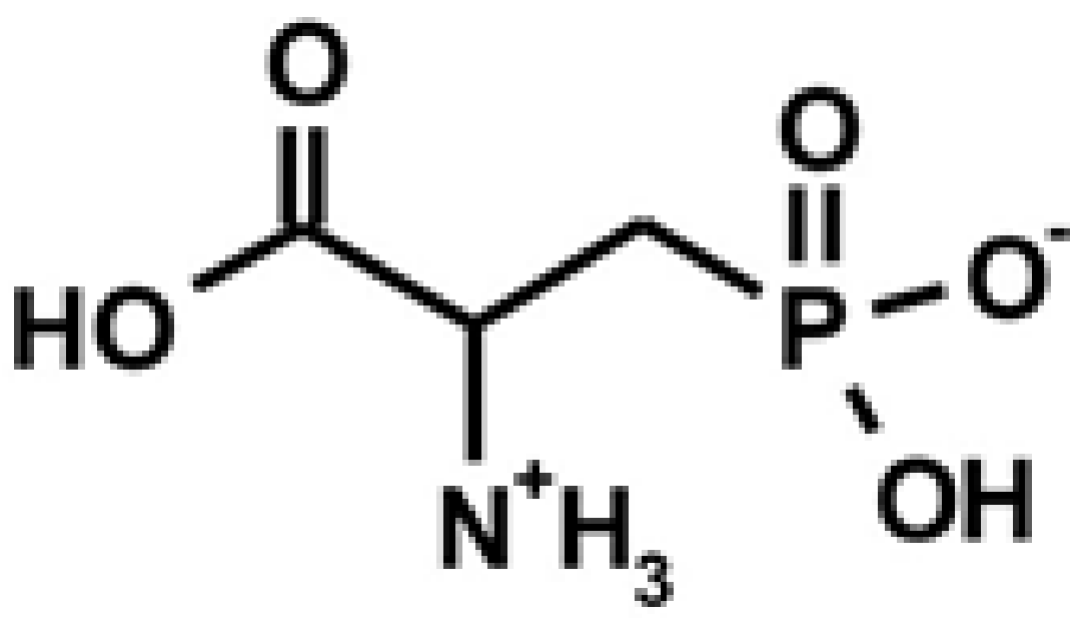	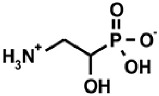	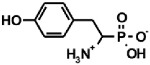	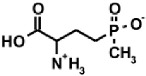
**β-Ala^P^ [4]**	Asp^β–P^ [5]	Iser^P^ [6]	Tyr^P^ [7]	Glu^−γ–P(Me)^ [8]
**Representative, biologically active aminophosphonates.**				
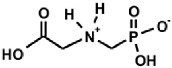	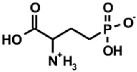	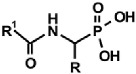	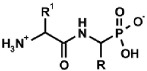	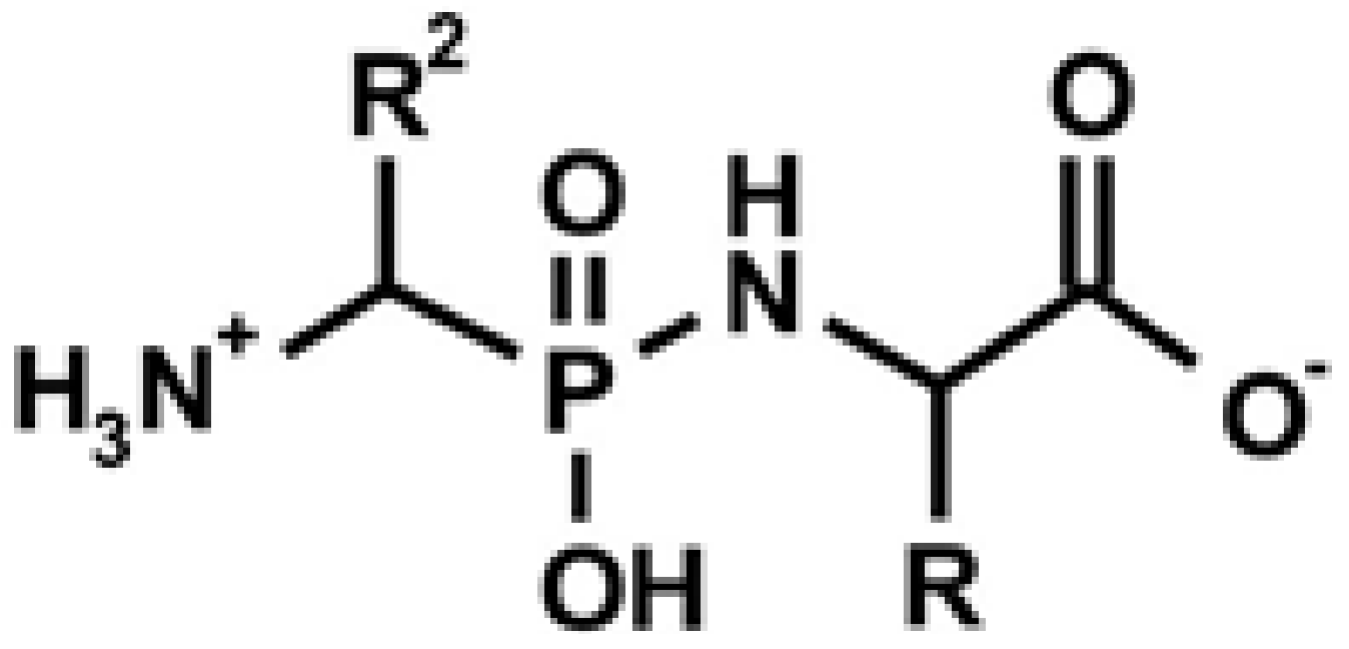
**PMG [9]**	Glu^γ–P^ [10]	(AC)-AA^P^[R=H, (AC)-Gly^P^] [11]	AA^C^-AA^P^(R=R^1^=Me, Ala-Ala^P^) [12]	AA^P^-AA^C^ [13,14]
**Abbreviation of AA^P^ and (AC)-AA^P^ follow the general rules elaborated by Kudzin et al. [15].**				

**Table 2:** ^31^P NMR chemical shifts of (AC)-AA^P^ and the corresponding adduct compounds (AC)(OH)-AA^P^ formed immediately after mixing the reaction mixture. (**A**) ^31^P NMR chemical shifts of Ac-AA^P^ and the corresponding adduct compounds Ac(OH)-AA^P^ formed. (**B**) ^31^P NMR chemical shifts of (AC)-Gly^P^ and the corresponding adduct compounds [AC(OH)]-Gly^P^ formed.

(**A**)										
	**Ac-Gly^P^**		**Ac-Ala^P^**		**Ac-Nva^P^**		**Ac-Val^P^**		**Ac-Pgl^P^**	
	Ac-Gly^P^	Ac(OH)-Gly^P^	Ac-Ala^P^	Ac(OH)-Ala^P^	Ac-Nva^P^	Ac(OH)-Nva^P^	Ac-Val^P^	Ac(OH)-Val^P^	Ac-Pgl^P^	Ac(OH)-Pgl^P^
δ	13.4	*12.8*	17.0	*16.5*	15.9	*15.2*	16.8	*16.1*	13.3	*12.7*
%	97.1	*2.9*	97.1	*2.9*		96.3	*3.7*	97.0	*3.0*	97.0	*3.0*
(**B**)										
	**Ac-Gly^P^**			**Prp-Gly^P^**				**Bz-Gly^P^**		
	Gly^P^	Ac-Gly^P^	Ac(OH)-Gly^P^	Gly^P^	Prp-Gly^P^	Prp(OH)-Gly^P^		Gly^P^	Bz-Gly^P^	Bz(OH)-Gly^P^
δ	19.1	13.4	12.8	19.3	13.4	12.8		19.3	13.5	13.2
%	-	97.1	2.9	-	98.3	1.7		-	99.5	<0.5

**Table 3:** Gibbs free energies ΔG (298 K) for reaction *1.1* in gas phase (kcal/mol).

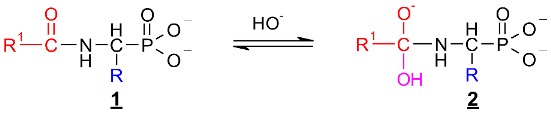				
(AC)-AA^P^ (1Xx)	R	*R*^1^*-C(O)-* (1Xx)		
		*CH*_3_*-* (1Ax)	*CF*_3_*-* (1Bx)	Ph- (1Cx)
**(AC)-Gly^P^ (1Xa)**	H-	139.43	123.16	126.87
**(AC)-Ala^P^ (1Xb)**	CH_3_-	135.19	119.26	124.82
**(AC)-Val^P^ (1Xc)**	(CH_3_)_2_CH-	133.61	117.48	132.28
**(AC)-Pgl^P^ (1Xd)**	Ph-	125.63	121.43	117.08
**(AC)-Phe^P^ (1Xe)**	PhCH_2_-	139.31	122.06	129.57

**Table 4:** Gibbs free energies ΔG (298 K) for reaction *1.1* in aqueous phase (kcal/mol).

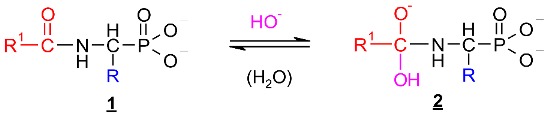				
(AC)-AA^P^ (1Xx)	R	*R*^1^*-C(O)-* (1Xx)		
		*CH*_3_*-* (1Ax)	*CF*_3_*-* (1Bx)	Ph- (1Cx)
**(AC)-Gly^P^ (1Xa)**	H-	26.70	11.77	25.70
**(AC)-Ala^P^ (1Xb)**	CH_3_-	26.26	10.54	25.45
**(AC)-Val^P^ (1Xc)**	(CH_3_)_2_CH-	26.15	10.90	26.57
**(AC)-Pgl^P^ (1Xd)**	Ph-	25.47	12.27	24.96
**(AC)-Phe^P^ (1Xe)**	PhCH_2_-	24.22	7.73	24.53

**Table 5:** Free energy barriers (ΔG^‡^ (298 K), kcal/mol) for OH^−^ addition (reaction *1.1*) in water.

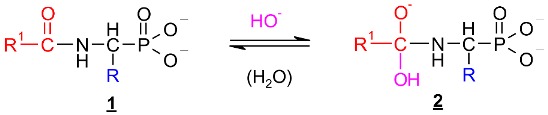				
(AC)-AA^P^ (1Xx)	R	*R*^1^*-C(O)**-* (1Xx)		
		*CH*_3_*-* (1Ax)	*CF*_3_*-* (1Bx)	Ph- (1Cx)
**(AC)-Gly^P^ (1Xa)**	H-	29.99	19.51	25.70
**(AC)-Ala^P^ (1Xb)**	CH_3_-	30.85	19.12	26.75
**(AC)-Val^P^ (1Xc)**	(CH_3_)_2_CH-	31.40	18.47	29.68
**(AC)-Pgl^P^ (1Xd)**	Ph-	28.57	17.60	28.44
**(AC)-Phe^P^ (1Xe)**	PhCH_2_-	28.85	18.66	30.23

**Table 6:** Gibbs free energy ΔG (298 K) for the reaction *1.2* in gas phase (kcal/mol).

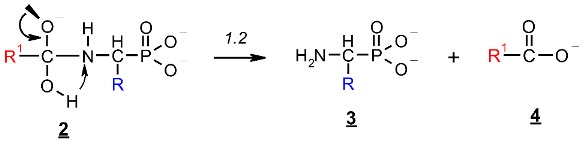				
(AC-OH)-AA^P^ (2-Xx)	R	*R*^1^*-C(O^−^)(OH)-* (2Xx)		
		*CH*_3_*-* (2Ax)	*CF*_3_*-* (2Bx)	Ph- (2Cx)
**(AC-OH)-Gly^P^ (2Xa)**	H-	−165.22	−153.75	−152.91
**(AC-OH)-Ala^P^ (2Xb)**	CH_3_-	−159.50	−149.37	−149.31
**(AC-OH)-Val^P^ (2Xc)**	(CH_3_)_2_CH-	−162.42	−152.74	−161.46
**(AC-OH)-Pgl^P^ (2Xd)**	Ph-	−157.24	−160.78	−150.14
**(AC-OH)-Phe^P^ (2Xe)**	PhCH_2_-	−168.00	−158.21	−159.56

**Table 7:** Gibbs free energy ΔG (298 K) for the reaction *1.2* in an aqueous phase (kcal/mol).

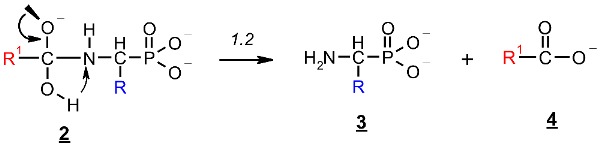				
(AC-OH)-AA^P^ (2-Xx)	R	*R*^1^*-C(O^−^)(OH)-* (2Xx)		
		*CH*_3_*-* (2Ax)	*CF*_3_*-* (2Bx)	Ph- (2Cx)
**(AC-OH)-Gly^P^ (2Xa)**	H-	−46.41	−40.32	−48.51
**(AC-OH)-Ala^P^ (2Xb)**	CH_3_-	−46.62	−38.58	−48.56
**(AC-OH)-Val^P^ (2Xc)**	(CH_3_)_2_CH-	−44.45	−37.28	−47.29
**(AC-OH)-Pgl^P^ (2Xd)**	Ph-	−45.60	−40.90	−47.44
**(AC-OH)-Phe^P^ (2Xe)**	PhCH_2_-	−47.74	−39.80	−50.89

**Table 8:** Free energy barriers (ΔG^‡^ (298 K), kcal/mol) for proton transfer/dissociation of the C-N bond in water (reaction *1.2*).

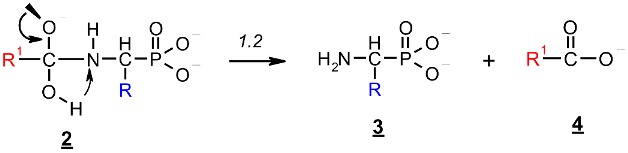				
(AC-OH)-AA^P^ (2-Xx)	R	*R*^1^*-C(O^−^)(OH)-* (2Xx)		
		*CH*_3_*-* (2Ax)	*CF*_3_*-* (2Bx)	Ph- (2Cx)
**(AC-OH)-Gly^P^ (2Xa)**	H-	20.76	21.54	16.91
**(AC-OH)-Ala^P^ (2Xb)**	CH_3_-	21.47	23.43	19.00
**(AC-OH)-Val^P^ (2Xc)**	(CH_3_)_2_CH-	23.73	24.91	20.56
**(AC-OH)-Pgl^P^ (2Xd)**	Ph-	20.05	21.74	18.51
**(AC-OH)-Phe^P^ (2Xe)**	PhCH_2_-	22.50	25.00	18.99

**Table 9:** Total Gibbs free energiesΔG^298^(*1.1*) + ΔG^298^(*1.2*) for the overall deacylation process in the aqueous phase and total Gibbs free energy barriers (ΔG^298^(*1.1*) + ΔG^‡^(*1.2*), in parentheses) (kcal/mol).

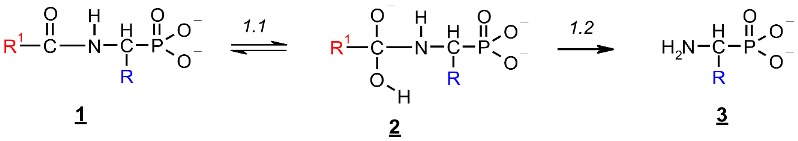				
(AC)-AA^P^ (1Xx)	R	*R*^1^*-C(O)-* (1Xx)		
		*CH*_3_*-* (1Ax)	*CF*_3_*-* (1Bx)	Ph- (1Cx)
**(AC)-Gly^P^ (1Xa)**	H-	−19.72 (47.5)	−28.56 (31.3)	−22.82 (42.6)
**(AC)-Ala^P^ (1Xb)**	CH_3_-	−20.36 (47.7)	−28.04 (34.0)	−23.11 (44.5)
**(AC)-Val^P^ (1Xc)**	(CH_3_)_2_CH-	−18.30 (49.9)	−26.38 (35.8)	−20.72 (47.1)
**(AC)-Pgl^P^ (1Xd)**	Ph-	−20.13 (45.5)	−28.63 (34.0)	−22.48 (43.5)
**(AC)-Phe^P^ (1Xe)**	PhCH_2_-	−23.53 (46.7)	−32.08 (32.7)	−26.36 (43.5)

**Table 10:** Structures, names and abbreviations of aminophosphonic acids and corresponding 1-(acylamino)phosphonic acids discussed in this work ^a^.

		**Name**	**Trivial Name**	**Abbr./(No)**	**Ref.**
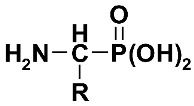		Aminoalkyl-phosphonic acid	Phosphono-Amino Acid	AA^P^ (**3**)	
**R**					
H		Aminomethyl-phosphonic acid	Phosphono-glycine	Gly^P^ (**3a**)	[64]
Me		1-Aminoethyl-phosphonic acid	Phosphono-alanine	Ala^P^ (**3b**)	[65]
iPr		1-Amino-1-methylethyl-phosphonic acid	Phosphono-valine	Val^P^ (**3c**)	
Ph		1-Amino-1-phenylmethyl-phosphonic acid	Phosphono-phenyl-glycine	Pgl^P^ (**3d**)	
PhCH_2_		1-Amino-1-phenylethyl-phosphonic acid	Phosphono-phenyl-alanine	Phe^P^ (**3e**)	
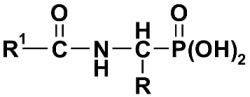		1-(Acylamino)alkyl-phosphonic acid	Acylamino-Phosphono Acid	(AC)-AA^P^ (**2**)	
**R^1^**	**R**				
CH_3_	H, Me, iPr, Ph, PhCH_2_	1-(Acetylamino)alkyl-phosphonic acid	Acetylamino-Phosphono Acid	Ac-AA^P^ (**2A**)	[36]
CH_3_	H	1-(Acetylamino)methyl-phosphonic acid	Phosphono-Acetylamino Glycine	Ac-Gly^P^ (**2Aa**)	
CH_3_	Ph	1-(Acetylamino)-1-phenyl-methyl-phosphonic acid	Phosphono-Acetyl-amino-PhenylGlycine	Ac-Pgl^P^ (**2Ad**)	
CF_3_	H, Me, iPr, Ph, PhCH_2_	1-(Trifluoroacetylamino)-alkyl-phosphonic acid	Phosphono-Trifluoroacetyl-amino Acid	TFA-AA^P^ (**2B**)	[37]
CF_3_	iPr	1-(Trifluoroacetylamino)-2-methylethyl-phosphonic acid	Phosphono-Trifluoroacetyl-Valine	TFA-Val^P^ (**2Bc**)	
Ph	H, Me, iPr, Ph, PhCH_2_	1-(Benzoylamino)alkyl-phosphonic acid	Phosphono-Benzoylamino Acid	Bz-AA^P^ (**2C**)	[36]
Ph	PhCH_2_	1-(Benzoylamino)-2-phenyl-ethyl-phosphonic acid	Phosphono-Benzoyl-Phenyl-alanine	Bz-Phe^P^ (**2Ce**)	

^a^ Applied names were in accordance with the IUPAC rules, and the abbreviations were in agreement with the general rules elaborated by Kudzin et al. [15].
